# Dominance of the *Enterocytozoon bieneusi* genotype BEB6 in red deer (*Cervus elaphus*) and Siberian roe deer (*Capreolus pygargus*) in China and a brief literature review

**DOI:** 10.1051/parasite/2017056

**Published:** 2017-12-21

**Authors:** Wei Zhao, Jianguang Wang, Ziyin Yang, Aiqin Liu

**Affiliations:** Department of Parasitology, Harbin Medical University, Harbin, Heilongjiang 150081 PR China

**Keywords:** *Enterocytozoon bieneusi*, Genotyping, ITS region, Red deer, Siberian roe deer, Zoonotic

## Abstract

*Enterocytozoon bieneusi* is the most frequently diagnosed microsporidian species in humans and is also found in a wide range of animals. It is considered to be an important but neglected zoonotic pathogen. With the development of deer bred in captivity, the number of deer has been increasing in recent years in China and there are more people involved in this work. The aims of this study were to determine prevalence and genotypes of *E.* *bieneusi* in red deer (*Cervus* *elaphus*) and Siberian roe deer (*Capreolus* *pygargus*), and to assess their potential zoonotic transmission. A total of 122 fecal specimens were collected from 104 red deer and 18 roe deer from three deer farms in Heilongjiang and Jilin Provinces, China. *Enterocytozoon* *bieneusi* was detected and genotyped by PCR and sequencing of the internal transcribed spacer (ITS) region of the rRNA gene. The average infection rate was 8.2% (10/122), with 7.7% (8/104) for red deer and 11.1% (2/18) for roe deer. Two genotypes were identified: a known genotype BEB6 (n = 9) and a novel genotype named HLJD-VI (n = 1). This is the first report of *E.* *bieneusi* infection in Siberian roe deer. The fact that genotype BEB6 was detected previously in one human case of microsporidiosis, and that genotype HLJD-VI fell into zoonotic group 1, suggest the possibility of transmission to humans. A brief review of *E.* *bieneusi* genotypes in deer worldwide shows that 40 genotypes have been found in seven deer species, with genotype BEB6 being predominant.

## Abstract (fr)

*Enterocytozoon* *bieneusi* est l'espèce de microsporidie la plus fréquemment diagnostiquée chez l'homme et se retrouve également chez un large éventail d'animaux. Elle est considérée comme un pathogène zoonotique important mais négligé. Avec le développement de l'élevage de cerfs en captivité, le nombre de cerfs a augmenté ces dernières années en Chine et il y a plus de gens impliqués dans ce travail. Les objectifs de cette étude étaient de déterminer la prévalence et les génotypes d'*E.* *bieneusi* chez le cerf élaphe (*Cervus* *elaphus*) et le chevreuil de Sibérie (*Capreolus* *pygargus*) et d'évaluer leur potentiel de transmission zoonotique. Au total, 122 spécimens de fèces ont été prélevés de 104 cerfs et 18 chevreuils de trois élevages de cerfs dans les provinces du Heilongjiang et du Jilin, en Chine. *Enterocytozoon* *bieneusi* a été détecté et génotypé par PCR et séquençage de la région de l'ITS du gène de l'ARNr. Le taux moyen d'infection était de 8,2 % (10/122), avec 7,7 % (8/104) pour le cerf et 11,1 % (2/18) pour le chevreuil. Deux génotypes ont été identifiés: un génotype connu, BEB6 (n = 9) et un nouveau génotype appelé HLJD-VI (n = 1). Ceci est le premier rapport d'infection par *E.* *bieneusi* chez le chevreuil sibérien. Le fait que le génotype BEB6 ait été détecté précédemment dans un cas humain de microsporidiose et que le génotype HLJD-VI appartiennent au groupe zoonotique 1 suggère la possibilité d'une transmission à l'homme. Une brève revue des génotypes d'*E.* *bieneusi* chez les cerfs dans le monde montre que 40 génotypes ont été trouvés chez sept espèces de cerfs, le génotype BEB6 étant prédominant.

## Introduction

The microsporidia, emerging as an important group of opportunistic pathogens, have a global distribution and at least 14 species are recognized as human pathogens [3]. Over 90% of human cases of microsporidiosis are caused by *Enterocytozoon* *bieneusi* [3]. *E.* *bieneusi* is one of the most significant causes of diarrheal diseases, especially in immunocompromized individuals [20]. *E.* *bieneusi* infections have been observed in many species of animals (mammals, birds and reptiles) [15,25]. Spores as the infective stage have been found in some environmental samples (water, soil and food) [2,16]. The epidemiology of human microsporidiosis caused by *E.* *bieneusi* is complex, and involves direct transmission (person-to-person and animal-to-person) and indirect transmission routes (through water, food and fomites contaminated with spores) [6].

Although light microscopy is commonly used in the diagnosis of microsporidia infections for stained clinical smears, especially for fecal samples, PCR-based molecular techniques have the ability to achieve an accurate diagnosis at species and genotype levels [9]. Currently, based on the high degree of diversity in the internal transcribed spacer (ITS) region of the ribosomal RNA (rRNA) gene within *E.* *bieneusi*, a standard method for genotyping *E.* *bieneusi* isolates has been established by sequence analysis of 243 bp of the ITS region [24]. To date, more than 240 ITS genotypes of *E.* *bieneusi* have been identified, while 33 of them are found in both humans and animals [20,25,30]. By phylogenetic analysis of nucleotide sequences of the ITS region, all the *E.* *bieneusi* genotypes can be unambiguously placed into nine distinct clades [12]. A large group (group 1) is composed of genotypes from both humans and animals, showing the zoonotic nature; the other eight groups (groups 2 to 9) mainly include genotypes from specific hosts or wastewater, showing host adaption to some extent [10]. To improve our understanding of the epidemiology of *E.* *bieneusi* and its populations, molecular epidemiological surveys should focus on genotyping *E.* *bieneusi* isolates from under-sampled animal hosts.

Deer are one of the more common wild ruminant mammals. The number of deer bred in captivity has been increasing due to their economic significance to humans, and there are more people involved in this work. It has been reported that these animals play an important role in the epidemiology of *E.* *bieneusi* in some countries or areas [6]. Red deer (*Cervus* *elaphus*) are one of the most common deer species worldwide, while Siberian roe deer (*Capreolus* *pygargus*) are found within the temperate zone of eastern Europe and Asia. The two deer species are commonly found in China, especially in the northeastern part. Currently, there are no reports available on *E.* *bieneusi* infection in these animals. The present study was performed to determine the prevalence, genetic diversity, and zoonotic potential of *E.* *bieneusi* in captive red deer and roe deer in northeastern China.

## Material and Methods

### Collection of fecal specimens

During the period from November 2015 to March 2016, 122 fecal samples were collected including 44 red deer from Great Hinggan Mountains in Heilongjiang Province, and 60 red deer and 18 Siberian roe deer from Liaoyang City in Liaoning Province. The present study protocol was reviewed and approved by the Research Ethics Committee and the Animal Ethical Committee of Harbin Medical University. The inclusion of the farm animals in the present study was authorized by the farm manager. One fresh fecal specimen (approximately 30 g) from each animal was collected from the ground immediately after defecation by sampling using a sterile disposal latex glove and then placing the sample into an individual plastic bag. Each individual was identified according to the ear tag, neck rope and body characteristics. All the fecal specimens were transported to the laboratory in a cooler with ice packs within 24 hours and stored in refrigerators at 4 °C prior to being used in molecular biological characterizations. Meanwhile, the age, gender and health status of each animal was recorded accordingly on the plastic bag. None of the animals showed clinical signs of illness at the time of sampling.

### DNA extraction and PCR amplification

After the fecal specimens were sieved, the filtrates were concentrated and washed three times with distilled water by centrifugation for 10 minutes at 1500 *g*. Genomic DNA was extracted from 180–200 mg washed fecal specimens using a QIAamp DNA Stool Mini Kit (QIAgen, Hilden, Germany) according to manufacturer-recommended procedures. DNA was eluted in 200 µL of AE buffer and stored at −20 °C prior to PCR analysis.

Each of the DNA specimens was analyzed for the presence of *E.* *bieneusi* by nested PCR amplifying a 390 bp nucleotide fragment of the rRNA gene including the 243 bp ITS region, and the primer sequences and the cycling parameters in nested PCR analysis were used as previously described [1]. TaKaRa Taq DNA polymerase (TaKaRa Bio Inc., Tokyo, Japan) was used in all the PCR amplifications. A negative control with no DNA added was included in all PCR tests. All the secondary PCR products were subjected to electrophoresis in a 1.5% agarose gel and visualized under UV by staining the gel with ethidium bromide.

### Nucleotide sequencing and analysis

All the secondary PCR products of the expected size were directly sequenced with primers used for the secondary PCR after being purified on an ABI PRISM 3730 XL DNA Analyzer by Sinogeno-max Biotechnology Co. Ltd. (Beijing, China), using the Big Dye Terminator v3.1 Cycle Sequencing Kit (Applied Biosystems, USA). Sequence accuracy was confirmed by two-directional sequencing and by sequencing at least two new PCR products for some DNA preparations, from which we obtained sequences with nucleotide substitutions, deletions or insertions. To determine *E.* *bieneusi* genotypes, nucleotide sequences obtained in the present study were aligned with each other and reference sequences downloaded from the GenBank database using the Basic Local Alignment Search Tool (BLAST) and Clustal X 1.83 (http://www.clustal.org/). If the sequences had 100% similarity with those from known genotypes, they would be given the first published name. If not, they were considered as novel genotypes. All the genotypes were named based on the 243 bp of the ITS gene region of *E.* *bieneusi*, according to the established nomenclature system [24].

### Phylogenetic analysis

To better present the diversity of all the genotypes obtained in the present study and the genetic relationship of novel ones to known ones, a comparison of the ITS region of all the nucleotide sequences obtained here and reference sequences previously published in GenBank was performed by implementing all the sequences obtained in Mega 5 software (http://www.megasoftware.net/). A neighbor-joining tree was generated based on the evolutionary distances calculated by the Kimura2-parameter model. The genogroup of the novel ITS genotype of *E.* *bieneusi* was designated. The confidence of the tree was assessed by bootstrap analysis using 1,000 replicates.

## Results and Discussion

The present study was conducted to determine whether *E.* *bieneusi* was present in 122 deer fecal specimens by amplifying the ITS region of the rRNA gene. Overall, ten of the samples were PCR-positive and confirmed to be infected with *E.* *bieneusi* by sequence analysis, with an average infection rate of 8.2% (10/122). The infection rates by *E.* *bieneusi* were 11.1% (2/18) in red deer and 7.7% (8/104) in Siberian roe deer (Table 1). For red deer, 5.0% (1/20) were positive for *E.* *bieneusi* in the young animals and 8.3% (7/84) in the adults, while 8.1% (6/74) were positive in females and 6.7% (2/30) in males (Table 2). None of the differences in infection rates were statistically significant either by gender (χ2 = 0.03, *p*>0.05) or by age (χ2 = 0.002, *p*>0.05). For Siberian roe deer, *E.* *bieneusi* was only found in two female adults (Table 2). This is the first report of *E.* *bieneusi* infection in Siberian roe deer. Molecular epidemiological data indicate that *E.* *bieneusi* has been found in all the deer species examined, including in the present study. To date, 137 deer-derived *E.* *bieneusi* isolates have been identified out of 852 deer. The infection rates by *E.* *bieneusi* varied from 7.1% to 75.0% in different deer species from different countries and areas (Table 3), with 75.0% in hog deer [17], 34.1% in Père David's deer [34], 16.8% in reindeer [19], 7.7–25.0% in red deer [17,37, this study], 7.1–50.0% in sika deer [17,33,37], 11.1% in Siberian roe deer (this study), and 12.2–32.5% in white-tail deer [10,26].

**Table 1:** Prevalence and genotypes of *Enterocytozoon bieneusi* in red deer and roe deer.

Deer species (Latin name)	Location	Province	No. positive/No. examined (%)	Genotype (n)
Red deer (*Cervus elaphus*)	Great Hinggan Mountains	Heilongjiang	3/44 (6.8)	BEB6 (2); HLJD-VI (1)
	Liaoyang	Liaoning	5/60 (8.3)	BEB6 (5)
Siberian roe deer (*Capreolus pygargus*)	Liaoyang	Liaoning	2/18 (11.1)	BEB6 (2)
Total			10/122 (8.2)	BEB6 (9); HLJD-VI (1)

**Table 2:** Prevalence and genotype distribution of *Enterocytozoon bieneusi* in red deer and roe deer by age and by gender.

Group		Red deer (*Cervus elaphus*)					Siberian roe deer (*Capreolus pygargus*)			
		
		No. examined		No. positive (%)		Genotype (n)	No. examined		No. positive (%)	Genotype (n)
Age	Young	20		1 (5.0)		BEB6 (1)	—		—	—
	Adult	84		7 (8.3)		BEB6 (6); HLJD-VI (1)	18		2 (11.1)	BEB6 (2)
Gender	Female	74		6 (8.1)		BEB6 (6)	16		2 (12.5)	BEB6 (2)
	Male	30		2 (6.7)		BEB6 (1); HLJD-VI (1)	2		0 (0)	—

**Table 3:** Prevalence and genotype distribution of *Enterocytozoon bieneusi* in natural infection of deer species worldwide.

Deer species	No. positive / No. examined (%)	Genotype (n) Group 1	Other Groups b	Reference
Hog deer (*Axis porcinus*)	3/4 (75.0)	—	BEB6 (2); CHS9(1)	[17]
Père David's deer (*Elaphurus davidianus*)	16/47 (34.0)	Type IV (4); EbpC (4); EbpA (4)	BEB6 (2); COS-I (1); COS-II (1)	[34]
Reindeer (*Rangifer tarandus*)	21/125 (16.8)	CHN-RD1(12); CHN-RD2(1); CHN-RD3(1); CHN-RD4(1); Peru6 (6)	—	[19]
Red deer (*Cervus elaphus*)	1/5 (20.0)	—	HLJD-V (1)	[37]
	1/4 (25.0)	—	BEB6 (1)	[17]
	8/104 (7.7)	HLJD-VI (1)	BEB6 (7)	This study
Sika deer (*Cervus nippon*)	28/86 (32.6)	HLJD-II (1); HLJD-III (1); HLJ-IV (1)	BEB6 (20); HLJD-I (1); HLJD-V (4)	[37]
	2/4 (50.0)	SCO3 (1)	BEB6 (1)	[17]
	23/326 (7.1)	CHN-DC-1 (1); KIN-1 (1); EbpC (1); JLD-2 (2); JLD-3 (1)	J (11); BEB6 (4); JLD-1 (2)	[33]
Siberian roe deer (*Capreolus pygargus*)	2/18 (11.1)	—	BEB6 (2)	This study
White-tail deer (*Odocoileus leucurus*)	6/49 (12.2)	WL18 (2); WL19 (2)	WL4 (2)	[10]
	26 a/80(32.5)	LW1 (1)	WL4 (11); I (7); J (1); DeerEb1-DeerEb13 (one each)	[26]
Total	137/852 (16.1)	HLJD-II (1); HLJD-III (1); HLJ-IV (2); Type IV (4); EbpC (5); EbpA (4); CHN-RD1(12); CHN-RD2(1); CHN-RD3(1); CHN-RD4(1); Peru6 (6); CHN-DC-1 (1); KIN-1 (1); JLD-2 (2); JLD-3 (1); WL18 (2); WL19 (2); LW1 (1); SCO3 (1)	CHS9(1); BEB6 (39);HLJD-V (5); HLJD-I (1); COS-I (1); COS-II (1); J (12); JLD-1 (2); WL4 (13); I (7); DeerEb1-DeerEb13 (one each)	

a : The numbers of genotypes are not consistent with the numbers of positives in White-tail deer due to mixed genotype infections [26].

b : All the genotypes in the fourth column are in Group 2 except WL4 belonging to Group 3.

In the present study, two genotypes were identified in red deer and Siberian roe deer by sequencing and multiple sequence alignment, including one known genotype (BEB6) and a novel one (HLJD-VI; KY681138), which had only one base difference (G → A at the 207 nucleotide site) with genotype Peru6 (JF927955). A total of 90.0% (9/10) of *E.* *bieneusi* isolates were identified as genotype BEB6. In contrast, genotype HLJD-VI was only found in a male adult red deer. Genotype BEB6 (SH5) was originally detected in cattle in the USA [5]. This genotype has also been found in a sample from a child, as well as in water samples in China [29,31]. To date, this genotype has been found in at least 15 animal species from five countries, and the majority of BEB6 isolates are identified from herbivore animals in China, including deer, sheep, goats, cattle and horses (Table 4). These findings suggest that this genotype has a broad host range and geographical distribution as well as a zoonotic nature. Meanwhile, these observations also support the theory of host-parasite co-evolution in *E.* *bieneusi*. Related hosts have the same *E.* *bieneusi* genotypes. Genotypes I and J are mainly found in cattle, sheep and goats [38,39].

**Table 4:** Host ranges and geographical distribution of *Enterocytozoon bieneusi* genotype BEB6.

Host		Isolate no.^a^	Reference
Mammal			
Human	Child	1	[29]
Carnivora	Cat (*Felis*)	2	[13]
Ruminant	Alpaca (*Vicugna pacos*)	1	[17]
	Cattle (*Bos taurus*)	2^b^	[5]
	Golden takins (*Budorcas taxicolor bedfordi*)	10	[36]
	Goat (*Capra hircus*)	126^c^	[7,11,21,27,38]
	Horse (*Equus ferus caballus*)	9	[23]
	Hog deer (*Axis porcinus*)	1	[17]
	Père David's deer (*Elaphurus davidianus*)	16	[34]
	Red deer (*Cervus elaphus*)	8	[17,37,This study]
	Sheep (*Ovis aries*)	384^d^	[8,18,27,28,32,38]
	Sika deer (*Cervus nippon*)	27	[17,33,37]
	Siberian roe deer (*Capreolus pygargus*)	2	This study
Rodent	Chinchillas (*Chinchilla lanigera*)	3	[22]
Nonhuman primate	Nonhuman primate	12	[4,12,14]
Bird			
	Duck (*Anas platyrhynchos domesticus*)	1	[35]
	Goose (*Anser domestica*)	4	[35]

a : The isolates without annotations are only from China.

b : The two isolates are from the USA.

c : 125 isolates are from China with one from Peru.

d : 341 isolates are from China, with 32 and 11 from Sweden and Brazil, respectively.

A high degree of genetic diversity has been observed within *E.* *bieneusi* in the ITS region of the rRNA gene. To date, at least 240 genotypes have been identified, including 40 genotypes identified in seven deer species (Table 3). Currently, it has been observed that 12 *E.* *bieneusi* genotypes identified in deer have been found in humans [20]. Meanwhile, most of the remaining genotypes from deer (12/28, 42.9%), including the novel genotype HLJD-VI, are clustered into group 1 by phylogenetic analysis (data not shown). Group 1 contains 94% of the published ITS sequences of *E.* *bieneusi,* and almost all the human-pathogenic genotypes are in this group [20]. Thus, although some genotypes of *E.* *bieneusi* from deer have not been found in humans, the risk of their zoonotic transmission should not be neglected.

## Conclusion

The present study reports the occurrence of *E.* *bieneusi* infection in Siberian roe deer for the first time. Based on the possibility of zoonotic transmission of deer-derived *E.* *bieneusi* isolates, and the fact that an increasing number of deer are bred in captivity, advice should be provide to those in contact with these animals, including farmers, breeders, veterinarians and animal handlers, to reduce the risk of zoonotic transmission of *E.* *bieneusi*.

## Competing interests

The authors declare that they have no competing interests.
